# Clinical Significance of Serum Protein Electrophoresis in Rapid Progression of Multiple Myeloma: A Case Report

**DOI:** 10.3390/clinpract16040081

**Published:** 2026-04-21

**Authors:** Silvia Iannelli, Melania Scarcella, Antonella Cusano, Federica Feleppa, Ylenia Pancione, Luigi Michele Pavone, Pasquale Cocchiaro

**Affiliations:** 1 PreClinic Pharmacology Services, 82030 Torrecuso, Italy; silvia.iannelli@unina.it (S.I.); cusano.antonella89@gmail.com (A.C.); 2 Department of Molecular Medicine and Medical Biotechnology, University of Naples Federico II, 80131 Naples, Italy; melania.scarcella@unina.it (M.S.); luigimichele.pavone@unina.it (L.M.P.); 3 Department of Anatomy Pathology, Azienda Ospedaliera San Pio, 82100 Benevento, Italy; federica.feleppa@aornsanpio.it; 4 Department of Clinical Pathology, Azienda Ospedaliera San Pio, 82100 Benevento, Italy; ylenia.pancione@aornsanpio.it

**Keywords:** serum protein electrophoresis, case report, multiple myeloma

## Abstract

**Background/Objectives**: Serum protein electrophoresis (SPE) is a widely used laboratory test for the detection and monitoring of monoclonal gammopathies, including multiple myeloma (MM). Although SPE is usually recommended in the presence of specific clinical or laboratory abnormalities, monoclonal gammopathies may occasionally develop rapidly and without typical symptoms. This case report aims to emphasize the diagnostic value of SPE in identifying an unexpected and fast-evolving monoclonal gammopathy. **Methods**: We report the clinical and laboratory eight-month follow-up of a 58-year-old male who initially underwent SPE for unrelated clinical conditions. Serial SPE analyses were performed using capillary zone electrophoresis. When abnormalities emerged, immunotyping and serum free light chain (FLC) assays were conducted. The diagnostic workup was completed with bone marrow aspiration, flow cytometry, and imaging studies according to current international diagnostic criteria. **Results**: The initial SPE (November 2023) showed a normal protein profile. After eight months, follow-up SPE revealed a prominent monoclonal spike in the gamma region (2.9 g/dL), associated with increased total serum proteins (91 g/L; range 64–82 g/L), elevated IgA levels (20.0 g/L; range 0.4–3.5 g/L), and a markedly abnormal κ/λ FLC ratio (54.00; range 0.31–1.56). Bone marrow analysis demonstrated >18% plasma cell infiltration, confirming the diagnosis of IgA-κ MM. The patient underwent standard therapy followed by autologous stem cell transplantation, achieving disease remission. **Conclusions**: This case highlights that clinically relevant monoclonal gammopathies may arise rapidly in the absence of classical diagnostic features. Routine SPE represents a cost-effective and accessible screening tool that can identify subtle protein abnormalities, prompting the timely use of more specific and invasive diagnostic procedures for aggressive plasma cell disorders.

## 1. Introduction

Serum protein electrophoresis (SPE) is a low-cost and simple laboratory test used in clinical practice to identify patients with monoclonal proteins, Multiple Myeloma (MM), immunoglobulin light-chain (AL) amyloidosis, lymphoid malignant diseases, and other serum protein disorders [1].

SPE is performed using capillary zone electrophoresis (CZE), a technique that separates proteins within narrow fused-silica capillaries under a high-voltage electric field. In CZE, analytes migrate through the capillary according to their charge-to-size ratio, and separation is further influenced by electroosmotic flow. Detection is carried out by continuous UV absorbance, producing a high-resolution electropherogram [2]. In CZE, human serum is exposed to an electrical current, which separates the proteins based on size and electrical charge into six fractions: albumin, alpha-1 (α1), alpha-2 (α2), beta-1 (β1), beta-2 (β2) and gamma (γ) [3]. CZE can differentiate healthy SPE patterns from disease conditions, such as the appearance of a monoclonal peak or band in the γ-region. In fact, interpretation of the SPE mainly focuses on the γ-region, which primarily contains immunoglobulins IgG, IgA, and IgM, since changes in this area are often associated with serious diseases. An increase in γ-globulins can produce different electrophoretic patterns. For example, monoclonal gammopathies like MM show a sharp and narrow peak (M-spike) in the γ-region. Conversely, an elevated and broad γ-region is typical of polyclonal gammopathies, which can occur in conditions such as chronic infections, autoimmune and connective tissue diseases [4,5], liver cirrhosis, Hodgkin’s disease, chronic lymphocytic leukemia, or amyloidosis. The γ band is decreased in agammaglobulinemia and hypogammaglobulinemia syndromes [6]. While various conditions can lead to an increase in the γ-region on serum protein electrophoresis, those that produce a distinct, spike-like peak within the γ-globulin zone are of clinical significance [7]. These are known as monoclonal gammopathies, a heterogeneous group of disorders characterized by clonal proliferation of plasma cells or B lymphocytes, resulting in the abnormal production of a monoclonal immunoglobulin (M protein) or free light chains (FLCs), typically detectable in the blood, urine, or both [8], and often indicates the presence of MM. To characterize the monoclonal nature of the peak, when an abnormal γ-region peak is detected, immunotyping (IT) is performed by overlaying antisera against heavy (IgG, IgA, IgM) and light (κ, λ) chains. The combination of SPE and IT thus provides both a screening and confirmatory approach in diagnosing a wide range of monoclonal gammopathies.

MM, which represents approximately 10–15% of all hematologic malignancies [9], is a clonal plasma cell neoplasm characterized by excessive production of monoclonal immunoglobulins (M proteins), usually exceeding 3 g/dL, along with ≥10% infiltration of clonal plasma cells in the bone marrow. Clinically, MM presents with a spectrum of symptoms, most notably bone pain, anemia, renal dysfunction, and hypercalcemia [10,11]. The M protein can act as a key tumor marker in MM and related monoclonal gammopathies, reflecting the underlying clonal plasma cell proliferation and production of a uniform immunoglobulin [12].

As the disease progresses, the clonal proliferation of malignant plasma cells within the bone marrow disrupts normal haematopoiesis and immunoglobulin production, leading to a range of clinical manifestations, including osteolytic lesions, focal bone resorption, and an increased risk of osteopenia, osteoporosis, and pathological fractures [13]. Despite significant advances in therapeutic strategies over the past two decades that have notably improved patient outcomes, MM remains an incurable malignancy, with a median overall survival of approximately 6 years following diagnosis [14]. Therefore, early and accurate diagnosis is critical to optimizing patient management. In this context, clinical laboratories play an essential role through the implementation of SPE and IT, which are fundamental in the early detection of monoclonal proteins and confirming diagnosis. This case presents an unusual and rarely documented rapid progression from a normal electrophoretic profile to overt MM within eight months, emphasizing the diagnostic relevance of routine SPE even in apparently asymptomatic patients. To our knowledge, such an acute transformation confirmed by serial laboratory and imaging findings has not been previously reported in the literature.

## 2. Materials and Methods

This study was conducted between November 2023 and July 2024. The patient was admitted to San Pio Hospital in Benevento.

The diagnostic workup for the patient included serial serum protein electrophoresis (SPE), immunotyping (IT), bone marrow aspiration, flow cytometry, and imaging studies (PET and MRI), according to current international diagnostic criteria.

### Serum Protein Electrophoresis and Immunotyping

Serum protein electrophoresis (SPE) was performed using capillary zone electrophoresis (CZE) on the Capillarys 3 Octa system (Sebia, Lisses, France). This technique separates serum proteins within fused-silica capillaries under a high-voltage electric field based on their charge-to-size ratio, with migration further influenced by electroosmotic flow.

Blood samples were collected in serum tubes, allowed to clot, and centrifuged at 3000 rpm for 10 min. Prior to analysis, serum was visually inspected for hemolysis, lipemia, and icterus. Separation was carried out in fused-silica capillaries (internal diameter 25–75 μm) using an alkaline buffer (pH ~8.6–10.0) with an applied voltage. Proteins were detected by continuous ultraviolet (UV) absorbance at a wavelength of 200–214 nm, generating a high-resolution electropherogram.

Under these analytical conditions, serum proteins were resolved into six major fractions: albumin, alpha-1 (α1), alpha-2 (α2), beta-1 (β1), beta-2 (β2), and gamma (γ). Electropherograms were processed using Phoresis Core software (version 9.32; Sebia, Lisses, France , which automatically integrated peak areas and provided quantitative results expressed as relative percentages and absolute concentrations (g/dL or g/L). IT was performed using the same system with antisera against IgG, IgA, IgM, κ, and λ chains to confirm monoclonal components.

Analytical performance, including precision and reproducibility, was verified according to internal laboratory quality control procedures, and each run included internal quality controls at both normal and pathological levels. CZE allows for the reliable detection of monoclonal components, which typically appear as narrow peaks in the γ-region. Consequently, clinical interpretation focused primarily on this region, which contains immunoglobulins (IgG, IgA, and IgM).

## 3. Results

In November 2023, a 58-year-old male patient was undergoing clinical follow-up for recurrent prostatitis. The patient’s past medical history was otherwise unremarkable, with no family history of hematological malignancies or significant comorbidities. At that time, he was not taking any long-term medications, except for occasional treatments for prostatic symptoms. As part of this evaluation, the clinician requested laboratory tests to rule out comorbidities and gain a more comprehensive clinical picture. Baseline laboratory values, including a complete blood count (CBC), renal function, and calcium levels, were all within reference ranges (Table 1).

**Figure 1:**
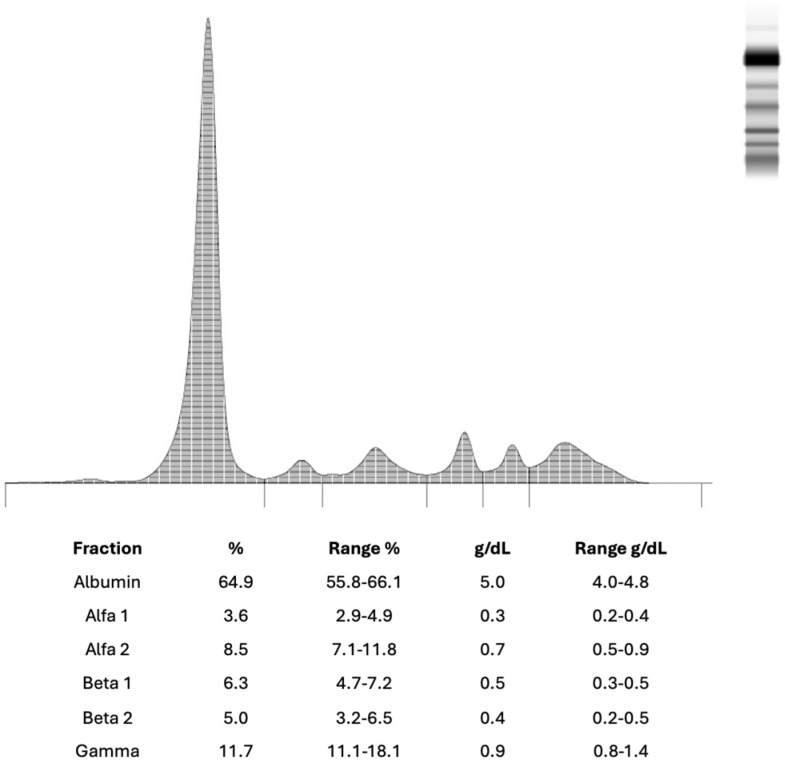
Normal serum protein electrophoresis pattern in a 58–year-old male patient from November 2023.

Although the patient did not exhibit CRAB (hypercalcaemia, renal involvement, anaemia, and bone lesions) criteria, serum protein electrophoresis (SPE) was ordered due to mild but persistent asthenia of unclear origin. The baseline SPE (November 2023) revealed a normal protein profile showed a normal protein profile (Figure 1), with a physiological distribution of serum proteins across all major fractions and no monoclonal spikes.

The patient showed no significant abnormalities in the SPE, except for an elevated prostate-specific antigen (PSA) of 11 μg/L (reference range: <4.0) and a free PSA of 2.52 μg/L, corresponding to a free/total PSA ratio of approximately 23%, which falls within the intermediate risk range for prostate cancer.

**Figure 2:**
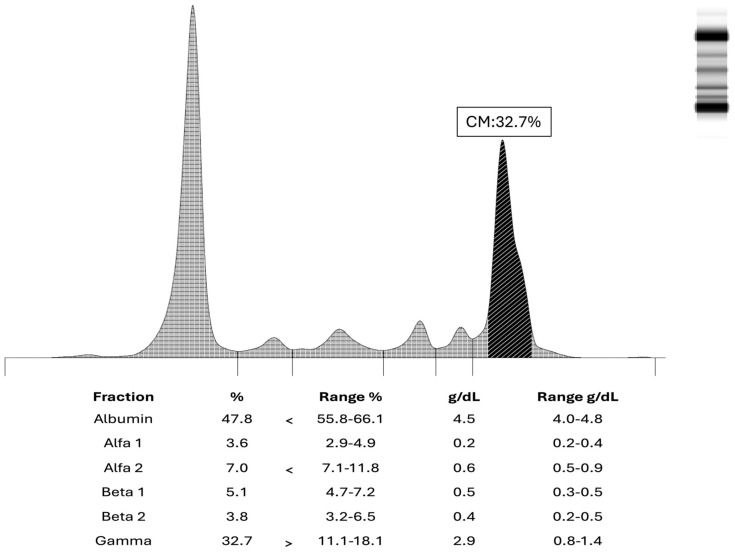
Pathological serum protein electrophoresis pattern in a 58-year-old male patient from July 2024.

The patient returned for follow-up eight months later, in July 2024. While prostatitis was again confirmed (PSA 9.83 μg/L; free PSA 2.24 μg/L), clinicians noted a significantly altered SPE pattern compared to the previous test (Figure 2). This follow-up analysis revealed a prominent monoclonal band in the gamma region (γ-globulin fraction: 32%; reference range: 11.1–18.8%) and an inverted albumin/gamma ratio of 0.99, strongly suggestive of a monoclonal gammopathy. These findings were accompanied by a notable increase in total serum proteins (91 g/L) and a stable but slightly increased serum calcium level (2.47 mmol/L), as detailed in Table 1.

Serum protein electrophoresis showed a monoclonal spike in the gamma region, indicative of a monoclonal gammopathy. No alterations were observed in the other fractions (albumin, alpha and beta).

Detailed laboratory investigations in July 2024 confirmed a markedly increased serum IgA (20.0 g/L; reference range: 0.4–3.5 g/L) and raised serum κ (kappa) light chains (7.76 g/L; reference range: 1.7–3.7 g/L). Haemoglobin (138 g/L; reference range: 120–170 g/L), serum calcium (2.47 mmol/L; reference range: 2.12–2.52 mmol/L) and creatinine levels (79.6 μmol/L; reference range: 53–106 μmol/L) remained stable and consistent with those detected in November 2023.

**Figure 3:**
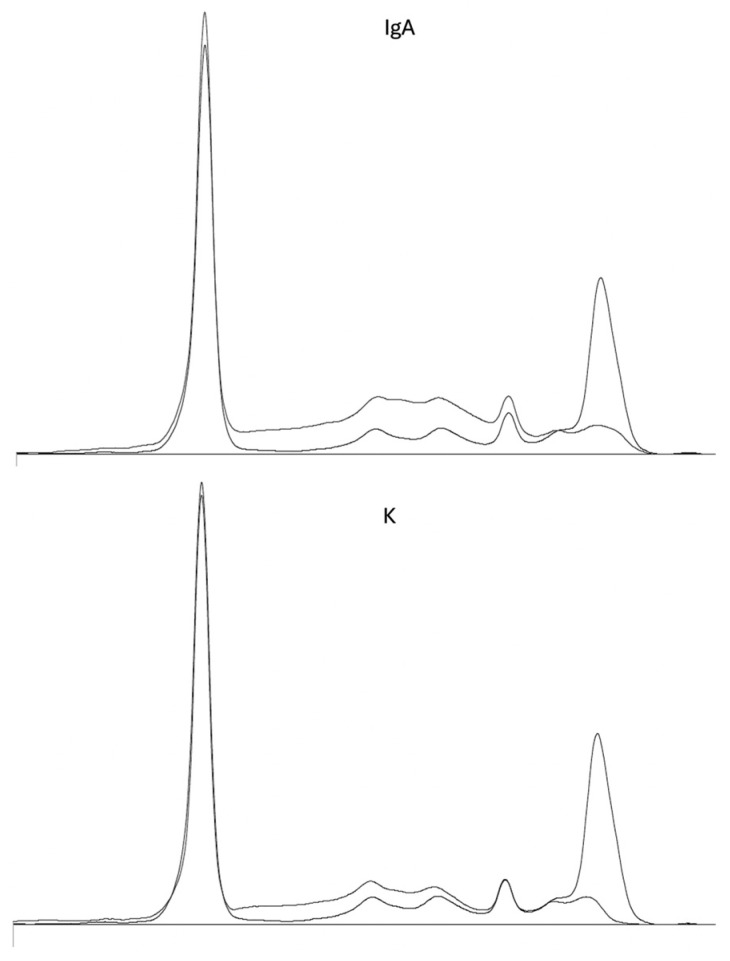
Immunotyping (IT) electropherogram of the patient’s serum (July 2024). The IT pattern revealed a distinct monoclonal band in the gamma region, which reacts specifically with anti-IgA (IgA) and anti-kappa (K) antibodies.

Immunotyping confirmed the nature of the monoclonal component as an IgA-κ type (Figure 3).

**Figure 4:**
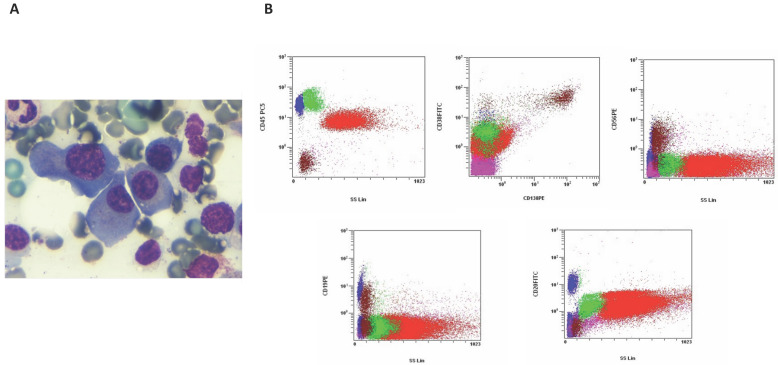
Bone marrow findings in the patient from July 2024. (**A**) Bone marrow aspirate shows a marked plasma cell infiltration (18%), indicative of a plasma cell disorder (May–Grünwald–Giemsa stain, 1000×). (**B**) Flow cytometry dot plots illustrating the gating strategy and immunophenotypic profile of plasma cells, characterized by CD45−, CD38+, CD56+, CD19+, and CD20− expression.

Serum free light chain analysis revealed a κ concentration of 0.584 g/L (reference range: 0.00670–0.02240) and a λ (lambda) concentration of 0.0108 g/L (reference range: 0.00830–0.02700), resulting in a markedly elevated κ/λ ratio of 54.00 (reference range: 0.31–1.56). To confirm the diagnosis, a bone marrow analysis was performed. Morphological examination of the bone marrow aspirate revealed over 18% of plasma cell infiltration (Figure 4A). Flow cytometry identified a pathological plasma cell population with the following immunophenotype: CD45−, CD38+, CD138+, CD56+, CD19+, CD28+, CD20−, CD33+, CD117+ and CD13− (Figure 4B).

**Figure 5:**
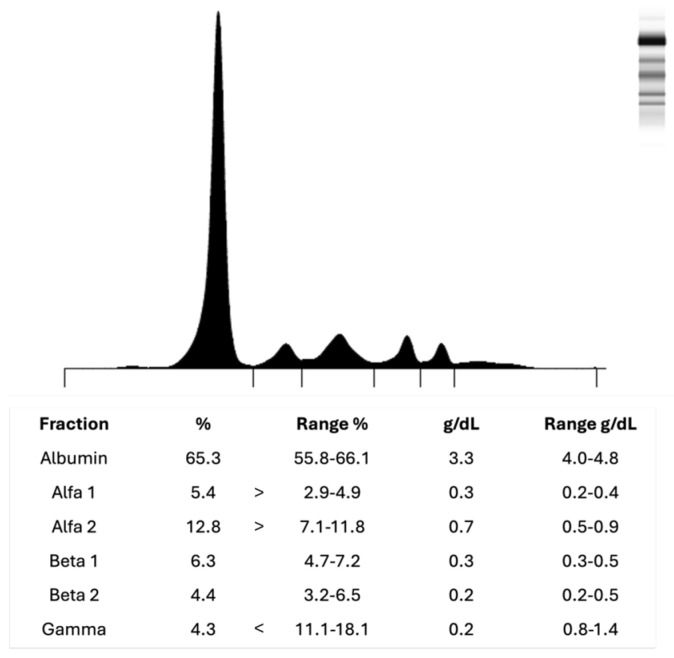
Serum protein electrophoresis (SPE) following autologous stem cell transplantation in a patient with multiple myeloma.

Imaging studies (PET and MRI) further supported the diagnosis of MM, in accordance with current diagnostic guidelines [10]. After the diagnosis of multiple myeloma, the patient underwent standard induction therapy followed by autologous stem cell transplantation, in accordance with current international treatment guidelines. Following this treatment, the disease entered remission, with persistent hypogammaglobulinemia, as shown by SPE results reported (Figure 5).

The analysis shows disease remission with persistent hypogammaglobulinemia after standard induction therapy and autologous stem cell transplantation, according to international treatment guidelines.

## 4. Discussion

Serum protein electrophoresis (SPE) is a crucial screening tool for detecting monoclonal gammopathies and other serum protein abnormalities [15]. It plays a significant role in the detection and monitoring of these conditions; its application in clinical diagnostics is also continually evolving with the newest Artificial Intelligence (AI) support [16]. Moreover, an emerging diagnostic approach, such as MALDI-TOF Mass spectrometry, has recently been evaluated to optimize the detection and identification of M protein. MALDI-TOF Mass spectrometry demonstrated 92% concordance with immunofixation electrophoresis (IFE) to identify immunoglobulin subtypes, even in cases where classical IFE results are ambiguous or negative [17].

At his initial presentation in November 2023, a 58-year-old male patient had unremarkable laboratory findings, except for elevated PSA levels, which were consistent with recurrent prostatitis. Notably, serum protein profiles, haemoglobin, calcium, and creatinine were within reference ranges, emphasizing that monoclonal gammopathies can emerge rapidly and silently in the absence of clinical symptoms [18]. At follow-up within eight months following the initial evaluation, the patient demonstrated a striking alteration in serum protein pattern, including a distinct monoclonal spike in the γ-region. Additionally, the albumin-to-gamma ratio decreased, in association with elevated total protein levels, and at the same time increased serum IgA and serum κ light chains. Identification of IgA-κ monoclonal component by IT, together with bone marrow biopsy analysis and imaging studies, confirmed the diagnosis of MM. This form of MM, characterized by the IgA-κ subtype, can often lead to significant renal and skeletal complications. Moreover, as observed in this report, it may present with a highly variable clinical course, ranging from an indolent to a more aggressive disease form [19].

This case highlights the asymptomatic or subtle disease progression of MM, even in patients being evaluated for unrelated conditions. The classical clinical features of MM, commonly summarized by the CRAB acronym (hypercalcaemia, renal involvement, anaemia and bone lesions) [20], were not observed in this patient, except for bone lesions identified only through imaging analysis.

The rapid evolution observed in this case highlights the clinical variability of MM and shows that significant monoclonal gammopathies can develop in patients without hematological features. Although some monoclonal gammopathies progress to malignancies at lower rates [21], our report may suggest that rapid progression is also possible, emphasizing the importance of careful clinical and laboratory follow-up when unexpected findings occur. Current guidelines recommend SPE in the evaluation of patients with unexplained anemia, bone pain, renal dysfunction, or hypercalcemia (CRAB criteria) [22]. However, this case highlights that significant gammopathies can emerge without these classic symptoms, emphasizing the usefulness of clinical vigilance. While SPE may not detect plasma cell dyscrasias earlier than high-sensitivity methods like flow cytometry or imaging, its clinical significance is rooted in its broad availability and low cost. In this case, the routine repetition of SPE served as the critical diagnostic trigger, justifying the subsequent use of more expensive and specific investigations like bone marrow aspiration and PET-MRI. Thus, SPE acts as a fundamental ‘gatekeeper’ in the diagnostic algorithm of Multiple Myeloma. The rapid progression from an undetectable monoclonal component to MM in less than 1 year, as observed in this patient, also suggests potential underlying biological aggressiveness, as well-documented for the IgA myelomas [23].

This case underscores the pivotal role of serum protein electrophoresis in the detection, diagnosis, and management of MM. It highlights the necessity of continuous collaboration between clinical laboratories and healthcare providers to ensure that subtle laboratory abnormalities are recognized, appropriately investigated, and translated into timely clinical interventions. Though a large study (iStopMM) was recently published [24], additional epidemiological investigations are still needed, specifically to better characterize and highlight rare or atypical cases such as the one presented here.

Ongoing research and refined screening protocols could enhance the early detection of aggressive forms of MM, especially in asymptomatic individuals [10].

## 5. Conclusions

This case study demonstrates the importance of SPE as a routine diagnostic test, particularly for the detection of MM and other gammopathies. Despite the absence of evident clinical symptoms, the patient showed a rapid progression from an undetectable monoclonal gammopathy to symptomatic MM within months, underscoring the potential aggressiveness of certain plasma cell disorders, particularly those involving IgA isotypes. We acknowledge the apparent lack of correlation between the first and second evaluations regarding the FLC and immunotyping results. However, at the time of the initial assessment, the electrophoretic pattern was interpreted as within normal limits, with no features suggestive of a monoclonal component. Consequently, there was no indication to proceed with immediate follow-up testing, such as immunotyping or serum free light chain (FLC) analysis.

Further research into integrating advanced diagnostic techniques with traditional electrophoretic methods is essential to improve diagnostic precision and patient outcomes. Nonetheless, SPE remains a rapid, informative, and reliable test in clinical practice.

In conclusion, this case indicates that these diseases, although rare, can develop quickly, highlighting the potential of SPE as a reliable and accessible primary screening tool. Developing integrated diagnostic strategies and conducting comprehensive further epidemiological studies are essential for better understanding and managing monoclonal gammopathies.

## Figures and Tables

**Table 1:** Clinical and laboratory parameters at baseline (November 2023) and follow-up (July 2024).

	November 2023	July 2024	Range	SI Units
White cells	6.34	5.83	4.00–10.80	10^9^/L
Red cells	5.58	5.20	4.2–6.10	10^12^/L
Haemoglobin	146	138	120–170	g/L
Platelets	204	190	130–400	10^9^/L
PSA	11.0	9.83	<4.00	μg/mL
Free PSA	2.52	2.24	<0.250	μg/mL
Total protein	77	91	64–82	g/L
Glucose	6.05	5.83	4.16–5.88	mmoL/L
Creatinine	70.7	79.6	53.0–103.4	μmoL/L
GOT	37	36	15–37	U/L
GPT	46	35	12–55	U/L
Calcium	2.27	2.47	2.1–2.522	mmoL/L

## Data Availability

The original contributions presented in this study are included in the article. Further inquiries can be directed to the corresponding author.

## Abbreviations

The following abbreviations are used in this manuscript:

CRAB	Hypercalcaemia, renal involvement, anaemia and bone lesions
CZE	Capillary zone electrophoresis
FLC	Free light chain
IFE	Immunofixation electrophoresis
IT	Immunotyping
MM	Multiple myeloma
SPE	Serum protein electrophoresis
